# Assessment of soft and hard tissue characteristics of ridge preservation at molar extraction sites with severe periodontitis: a randomized controlled trial

**DOI:** 10.1186/s12903-022-02544-0

**Published:** 2022-11-17

**Authors:** Haoyun Zhang, Yiping Wei, Tao Xu, Min Zhen, Cui Wang, Ziyao Han, Wenjie Hu, Kwok-Hung Chung

**Affiliations:** 1 Department of Periodontology, Peking University School and Hospital of Stomatology, National Center of Stomatology, National Clinical Research Center for Oral Diseases, National Engineering Laboratory for Digital and Material Technology of Stomatology, NHC Research Center of Engineering and Technology for Computerized Dentistry, 22 Zhongguancun S Ave, Haidian District, 100081 Beijing, China; 2 Department of Emergency, Peking University School and Hospital of Stomatology, National Center of Stomatology, National Clinical Research Center for Oral Diseases, National Engineering Laboratory for Digital and Material Technology of Stomatology, NHC Research Center of Engineering and Technology for Computerized Dentistry, Beijing, China; 3 Department of Restorative Dentistry, School of Dentistry, University of Washington, Seattle, WA USA

**Keywords:** Severe periodontitis, Tooth extraction, Alveolar ridge preservation, Natural healing, Cone beam-computed tomography, Soft tissue contour, Histological evaluation

## Abstract

**Background:**

Changes in alveolar bone dimension after tooth extraction may affect placement of the subsequent implant, resulting in ridge deficiency that can adversely impact long-term implant stability or aesthetics. Alveolar ridge preservation (ARP) was effective in reducing the amount of ridge resorption following tooth extraction. There is sparse evidence regarding the benefit of ARP at periodontally compromised molar extraction sockets. This study will be a randomized trial to assess the soft tissue contour, radiographical, and histological changes of ARP at molar extraction sites in order to compare severe periodontitis cases with natural healing results and determine the most beneficial and least traumatic clinical treatment for such patients.

**Methods:**

This research is designed as a two-group parallel randomized controlled trial. The total number of tooth extraction sites will be 70 after calculation with power analysis. Teeth will be randomly assigned to two groups with the test group conducting ridge preservation and the control group healing naturally. Periodontal examination, cone beam-computed tomography (CBCT) data, and stereolithographic (STL) files obtained by intraoral scanning will be collected through the follow-up period, and bone biopsy samples would be obtained during implant surgery. The primary outcomes are the vertical and horizontal change of alveolar ridge measured on CBCT images, soft tissue contour changes evaluated by superimposing the digital impressions, alterations of mucosa thickness (as measured by superimposing the CBCT data and STL files), histological features of implant sites and periodontal parameter changes. The secondary outcomes are patient-reported post-operative reaction and conditions of simultaneous bone graft or sinus lifting procedures during implantation.

**Discussion:**

This study will provide information about hard and soft tissue dimension changes and histomorphology evaluation following ARP and natural healing in periodontally compromised molar sites, which may contribute to complement the missing information of ARP at periodontally compromised molar extraction sockets.

**Trial registration:**

Chinese Clinical Trial Register (ChiCTR) ChiCTR2200056335. Registered on February 4, 2022, Version 1.0.

## Background

Periodontitis is a chronic inflammatory condition which affects the tissues surrounding and supporting the teeth. Globally, severe periodontitis was the sixth-most prevalent health condition, affecting about 10.8% of population [1]. In recent decades, the total burden of periodontitis has increased [2], and it has become the main cause of tooth loss in adults [3]. Molar plays an important role in oral function, while it presents a challenge for periodontal treatment [4], and shows a higher risk for tooth loss due to its anatomy and position [5].

A systematic review reported that a certain amount of alveolar bone resorption occurs after natural healing, and more reduction can be expected in molar extraction sites [6]. This dimensional change of the alveolar ridge may affect the subsequent implant procedure, resulting in ridge deficiencies that can adversely impact long-term implant stability or aesthetics [7–9], necessitating additional reconstructive surgery [10, 11]. A consensus report concluded that alveolar ridge preservation (ARP) attenuated the bone resorption following tooth extraction [12]. Moreover, the indications for ARP procedures have been widening from anterior intact extraction socket to infected molar extraction sockets [13–16].

Regarding infected molar extraction, Zhao et al. [17] has confirmed that the molar extraction sockets with advanced periodontitis did resorb and change in various sites, especially at the buccal wall of the socket. Fok et al. [18] reported that prosthetically driven implants planning at first molar extraction sites due to terminal periodontitis poses greater challenge to rehabilitation, often requiring advanced augmentation procedures and sinus augmentation. Nevertheless, Kim et al. [19] revealed that ridge preservation at periodontally compromised sockets was safe and effective in reducing bone resorption. Wei et al. [20] concluded that ridge preservation could decrease the necessity of further regenerative procedures at maxillary molars with severe periodontitis during implant placement compared to natural healing sockets.

In measurement technique, most of the previous investigations usually used cone beam-computed tomography (CBCT) or peri-apical film to evaluate the change of width and height of the alveolar socket received ARP [13, 20–23]. Only a few studies reported on the three-dimensional soft tissue profile changes using intraoral scanning technique for infected molar extraction sockets [24–26].

Histological analysis is valuable in assessing and determining the clinical application of ARP, since this procedure is often related to a following dental implant therapy that requires sufficient osseointegration within genuine bone tissue [27]. Previous studies showed ridge preservation using deproteinized bovine bone mineral (DBBM) had a lower amount of new bone formation in comparison with natural healing after a follow-up period of 3–6 months [28]. Further studies with longer healing time are needed for optimizing the clinical effects of ridge preservation in periodontally compromised sockets.

Although many previous studies have confirmed the effectiveness of ARP, few studies have been conducted in periodontally compromised molar extraction sites. Soft tissues surrounding implants have significant impact on esthetics and long-term health for dental implant therapy, but the comparison between natural healing and ARP in this area is also lacking. The different histological manifestations and the effect of implant therapy for these methods are also worthy of further exploration. Therefore, this randomized controlled trial is designed to compare the efficacy of ARP and natural healing at molar extraction sites in cases with severe periodontitis. This study is expected to determine the most beneficial and least traumatic clinical treatment for such patients. The following aspects will be compared, (i) soft tissue contour and mucosa thickness change from the baseline till at least 1 year after implant loading, (ii) radiographical evaluation for vertical and horizontal change of alveolar ridge from baseline to 6 months after atraumatic tooth extraction and ARP, (iii) histological composition after 6-month healing period in /tooth extraction sites with ARP, (iv) periodontal parameter changes in the follow-up period, (v) patient-reported post-operative reaction, and (vi) finally the need of simultaneous bone graft or sinus lifting procedures during implant placement procedure.

## Methods

**Fig. 1:**
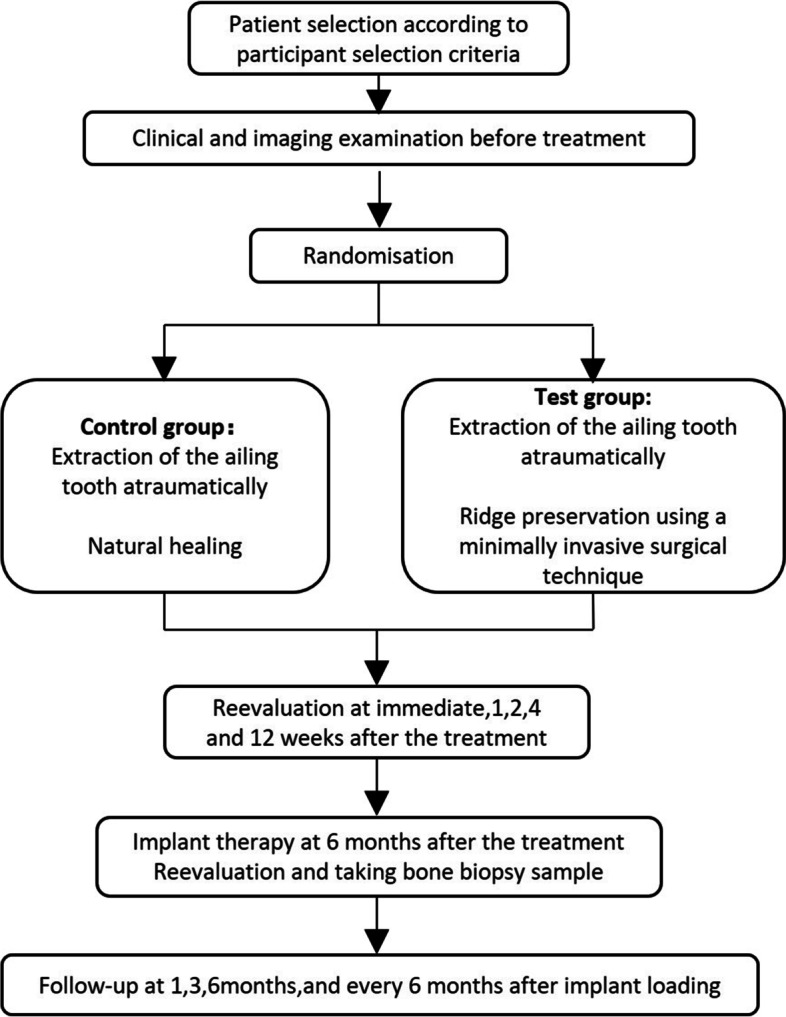
Framework of this trial

**Fig. 2:**
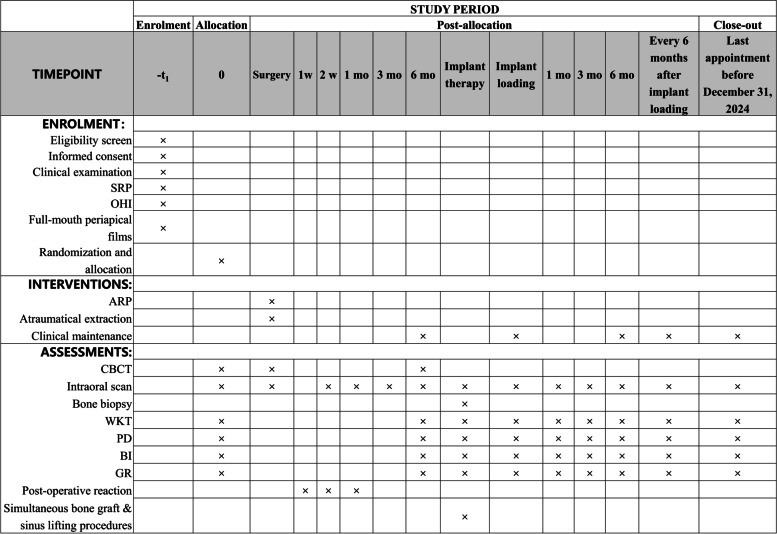
Standard Protocol Items: Recommendations for Interventional Trials (SPIRIT) Figure: schedule of enrolment, interventions and assessments. SRP: scaling and root planning; OHI: Oral hygiene instruction; ARP: Alveolar ridge preservation; CBCT: Cone beam-computed tomography; WKT: Width of keratinized tissue; PD: Probing depth; BI: Bleeding index; GR: Gingival recession

This research is a two-group parallel randomized controlled trial (RCT) to provide soft tissue contour, radiographical, and histological evaluation of the efficacy of ARP in 70 molar extraction sites with severe periodontitis. Figure 1 shows the framework of this trial. The design and report of this protocol follow the Standard Protocol Items: Recommendations for Interventional Trials (SPIRIT) statement [29], and the schedule of this study is shown in Fig. 2.

### Setting

This trial will be undertaken at the Peking University School and Hospital of Stomatology (Beijing, China). Before the trial takes place, written informed consent will be obtained from all participants.

### Participants

If a patient has unsalvageable molars as a consequence of severe periodontal disease and considers implant-retained prostheses for the extraction sockets, the patient will be suggested and reclused in this study.

The included patient should meet the following criteria:

1. Age more than 25 years
2. Good compliance and good oral hygiene
3. Diagnosed with stage III/IV periodontitis [30]
4. Presence of at least one hopeless molar with severe bone loss requiring extraction
5. At least two socket walls beyond the apex or ≥ 3 mm of the extraction socket
6. At least one adjacent tooth at the proximal region.

The exclusion criteria are as followed:

1. Pregnancy or lactation
2. Patients taking medications or having disease that would complicate bone healing
3. Patients with surgical contraindications, such as uncontrolled hypertension or diabetes mellitus
4. Patients with history of head and neck radiotherapy
5. Smoking more than 10 cigarettes per day
6. Absence of both adjacent teeth
7. Teeth with ongoing acute pathology
8. Teeth extracted due to caries, endodontic failures or fractured teeth.

### Criteria for discontinuing

The exclusion criteria will be checked regularly during the study period. If the participant meets any of the exclusion criteria during the follow-up period of this study, she/he will be excluded from the study. If the participant shows poor compliance or decides to withdraw the consent due to any reason, the participation will be terminated.

### Strategies to improve adherence

All time points in this study procedure will be matched with routine maintenance visits as far as possible to ensure the adherence to this clinical trial.

### Randomizing

A series of random numbers will be generated using Excel 2021 (Microsoft Corp., Redmond, WA, USA), written on letter paper and sealed in sequentially numbered, sealed, opaque envelopes. All the patients will be assigned to two groups by simple randomization with the rate of 1:1: the test group with alveolar ridge preservation, and the control group with natural healing. All the surgical procedures will be performed by an experienced clinician, and the other clinician will conduct the clinical examination for the patients. Another two members will take charge of intraoral scanning, assessing the radiological examinations and completing statistical analysis.

### Blinding

Blinding is not suitable for this trial. For the participants, they can distinguish which group they are in after experiencing different surgical procedures and post-operative treatment, so can the surgeons. For the assessors, they can also differentiate two groups from different imaging manifestations.

### Intervention

The enrolled patients will receive clinical and radiological examinations. They will receive routine scaling and root planing, and oral hygiene instruction at least 1 week before the surgery. All the patients will also receive prophylactic antibiotic medicine (Amoxicillin, 1 g or Erythromycin 300 mg if allergic to Penicillin) and anti-inflammatory drug (Ibuprofen 300 mg) 1 h prior to the surgical procedures described previously [31].

For control group:

1. An internal bevel incision will be performed from 0.5–1.0 mm below the buccal and lingual gingival margin to the bone crest.
2. The selected tooth will be extracted atraumatically [31].
3. The socket will be examined carefully and debrided thoroughly (using P24G Periosteal Elevator and CL86 Lucas Surgical Curette) without damaging the socket walls.

For test group:

1. The steps mentioned above are the same.
2. A full thickness flap will be elevated to expose 2 mm of the bone crest.
3. The socket will be filled with DBBM (Bio-Oss, Geistlich Pharma AG, Wolhusen, Switzerland), and a resorbable membrane (Bio-Gide, Geistlich Pharma AG, Wolhusen, Switzerland) will be used to cover the socket completely with 2 mm extending over the crest.
4. Then the socket will be covered by collagen sponge (Wuxi Biot Biology Technology Co.,Ltd., Wuxi, China).
5. A cross-mattress tension-free 5–0 suture will be placed over the site.

After the surgical procedures, patients will be prescribed to take the antibiotic three times daily for 7 days and ibuprofen (300 mg twice daily for 3–5 days) if needed. Patients will be required to perform regular tooth brushing in the rest of the mouth and oral rinse with 0.12% chlorhexidine solution for the 4-week post-surgery. The sutures will be removed 2 weeks after the surgery, and all the patients will be recalled at 4 weeks after the surgical to evaluate the healing status of the surgery site.

During the whole follow-up period from baseline to the last appointment, all the participants will receive regular maintenance therapy.

### Outcome measures

#### Baseline examination (Fig. *3* )

**Fig. 3:**
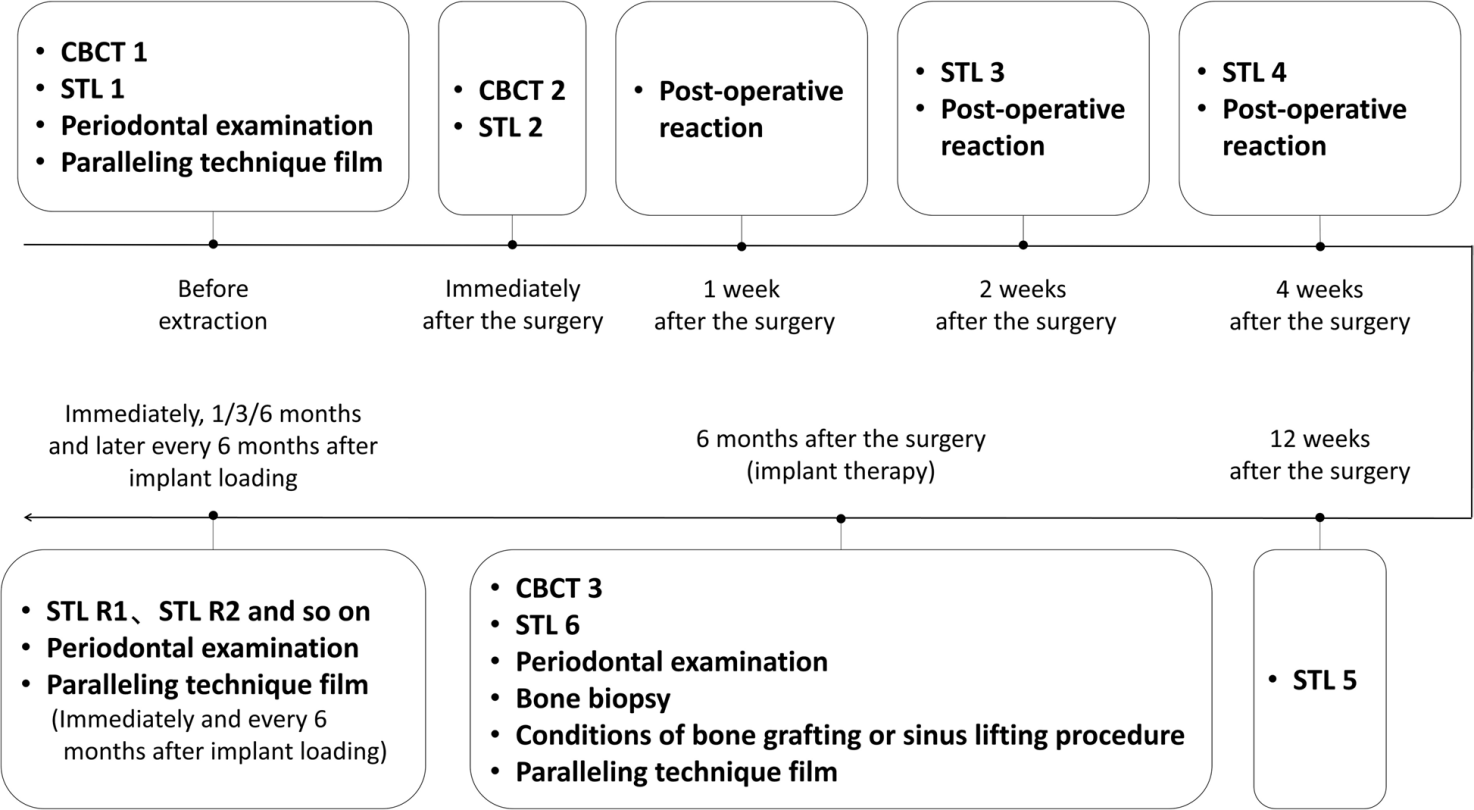
Flowchart of data collection during follow-up period

At baseline (before tooth extraction), the following data will be collected:

1. Full-mouth peri-apical films,
2. CBCT images (CBCT 1): CBCT images will be taken by CBCT machines (NewTom VG; Aperio Services, Italy) at a resolution of 0.125 mm with field of view size 10 × 10 cm (exposure time: 3.6 s, 110 kV, 5 mA). It will be used for assessing anatomical conditions, including the degree of bone loss and the position of maxillary sinus/mandibular canal.
3. Stereolithography (STL) files (STL 1): intraoral scans will be taken using a TRIOS intraoral scanner (3Shape TRIOS Color, TRC, 3Shape, Denmark) to obtain a digital impression of the target area and generate an STL file, which include the selected tooth and relevant quadrant. Soft tissues contained in this digital impression will extend over the mucogingival junction.
4. Periodontal parameters: the periodontal conditions of the ailing tooth and adjacent teeth will be assessed by measuring probing depth (PD), gingival recession (GR) and bleeding index (BI). The width of keratinized tissue (WKT) will be measured at mid-buccal aspect from the mucogingival junction to the gingival margin of the teeth. Data will be round down to the nearest 0.5 mm, and they will be collected using an UNC-15 probe (Hu-Friedy, Chicago, IL, USA).

#### Examination immediately after the intervention for both groups (Fig. 3)

1. CBCT images (CBCT 2): it will be used for superimposition and measurement. And it will be illustrated in informed consent.
2. STL files (STL 2)

#### Examinations during the follow-ups before implant therapy (Fig. 3)

1. STL files (STL 3–6): the patients will be recalled at 2, 4, 12 weeks and 6 months after the surgery, and intraoral scans will be performed.
2. CBCT images (CBCT 3): CBCT examination will be performed 6 months after the surgery.
3. Post-operative reaction: the patients will be recalled at 1, 2 and 4 weeks after the surgery to report post-operative reaction, such as pain, erythema, swelling and paranesthesia.
4. Paralleling technique films: films will be taken 6 months after the surgery.
5. Periodontal parameters: at 6 months after the surgery, the periodontal examination indicators (PD, GR, BI and WKT) will be recorded for the adjacent teeth, and WKT of extraction area will also be measured at the buccal aspect from the central of the (expected) implant position to the buccal mucogingival junction [32].

#### Examination during implant therapy (Fig. 3)

The implant therapy will be performed around 6 months after ARP/tooth extraction.

1. Bone biopsy: during the implant placement procedure, a cylindrical bone block with a diameter of about 2 mm and length of about 5 mm will be taken out using a trephine drill (Institute Straumann AG, Basel, Switzerland). The block will be fixated in 4% formaldehyde solution at room temperature for at least one day. Then, they will receive decalcification for 3 weeks in 4.1% disodium ethylene-diamino-tetra-acetic acid-solution, the solution will be changed every 24 h. After hydration, they will be dehydrated in increasing concentrations of ethanol (from 70 to 100%), embedded in paraffin, and cut into sections with a thickness of 4 µm. Serial sagittal sections will be stained with hematoxylin–eosin. Representative regions of interest (ROIs) will be localized in the center, apically and coronally of the sample [33]. Histologic slides will be observed under light microscopes and digital images will be obtained by scanning at a magnification of × 200 for subsequent histomorphometric analysis. For the two groups, the histological composition in terms of new bone formation, residual graft particles and connective tissue will be evaluated and compared.
2. Simultaneous bone graft & sinus lifting procedures: the conditions of bone grafting and sinus lifting during implant therapy will be recorded.

#### Examination during the follow-ups after implant restoration (Fig. 3)

After 6-month period of osseointegration, the superstructures will be connected to the implants. Participants will be recalled at immediately, 1-, 3-, and 6-month and then every 6 months after implant loading. During this period, the following data will be collected:

1. STL files (STL R1, R2, and so on): intraoral scans will be performed at every appointment.
2. Periodontal parameters: the periodontal examination indicators (PD, GR, BI and WKT) will be recorded for the implant and adjacent teeth at every appointment.
3. Paralleling technique films: the films will be collected immediately and every 6 months after implant loading, and they will be used to evaluate the marginal bone loss (MBL).

#### Superimposition and measurement of CBCT images and digital impressions

1. CBCT images: Digital Imaging and Communications in Medicine (DICOM) data will be generated and transferred to a volumetric imaging software (Mimics 21.0, Materialise, Leuven, Belgium). Residual buccal and lingual (or palatal) plate thicknesses and height of residual socket walls can be measured and recorded with vertical/horizontal reference lines and dots on CBCT 2. After Superimposition of CBCT 2 and CBCT 3, horizontal width and vertical height changes at target sites will be calculated; details are discussed in the previous literature [13, 17].
2. STL files: these data will be generated and imported to Geomagic Studio 2021 (3D Systems Inc., Rock Hill, SC, USA). Superimposition of STL 1 and STL files for subsequent stages (STL 2–6, STL R1, and so on) will be completed for the selected areas, using identified landmarks such as cusps of adjacent teeth. After superimposition, the impressions can be aligned and manually checked for perfect matching, and changes of the contour lines of alveolar ridge can be observed and measured.
3. CBCT data and STL files: As described in previous literature [25], CBCT 2 and STL 3 will be superimposed in Geomagic Studio 2021 (3D Systems Inc., Rock Hill, SC, USA) using three or more landmarks of adjacent teeth and so will CBCT 3 and STL 6. The vertical thickness of the crestal mucosa and horizontal thickness of the buccal and palatal/lingual mucosa and their alterations will be measured.

### Sample size calculation

The sample size was calculated using the Power Analysis and Sample Size (PASS) (version 15.0, NCSS, LLC, East Kaysville, Utah, USA) based on a two-sided alpha of 0.05 and power of 0.8. According to previous literature [13], the ridge width changes in one site (at 1 mm apically from the top of the alveolar crest in central buccal) of ridge preservation group and natural healing group were 1.46 mm and -0.70 mm, respectively. And the SD were 3.54 mm and 2.28 mm. After the calculation, the minimal sample size was 30 extraction sites for each group. Considering 15% non-response rate, 35 extraction sites will be included in this study for each group. Totally, 70 extraction sites will be needed.

### Statistical analysis

Statistical analysis will be performed using the SPSS 26.0 software package (SPSS Inc., Chicago, IL, USA). Descriptive data will be reported as mean ± standard deviations. A Shapiro–Wilk test will be applied to test for normal distribution of the sample for each variable. The paired t-test or Wilcoxon signed rank sum test when the data are not normally distributed will be applied to detect changes of soft and hard tissue before and 6 months after surgery. Independent samples t tests will be performed to compare parameter means between control and test groups. A non-parametric Mann–Whitney U test will be used if parameters are not normally distributed. Baseline characteristics of the patients who are lost to follow-up and completed this study will be compared. For the missing outcome data, K-means clustering method will be used for imputation. The level of significance was set at α = 0.05.

### Research ethics committee/institutional review board (REC/IRB) approval

This trial will be performed in strict accordance with the World Medical Association Declaration of Helsinki. The study protocol and written documents has been reviewed by the Ethics Committee of Peking University School and Hospital of Stomatology and received approval (PKUSSIRB-202170189). Written informed consent to participate will be obtained from all participants.

### Withdrawal

Participants will be informed that they can quit this study at any time without influencing their treatment process in the future.

## Discussion

This is a two-group parallel randomised controlled trial to compare the multi-dimensional efficacy of ARP and natural healing at molar extraction sites with severe periodontitis. Since Kim et al. [19] showed the safety and effectiveness of ridge preservation at periodontally compromised sockets, an increasing number of studies has reported that ridge preservation in these sites was effective in reducing the amount of ridge resorption [13, 14, 20]. Wei et al. [20] evaluated ridge preservation in the maxillary molar extraction sockets with severe periodontitis, and concluded that ARP could decrease the necessity of advanced regenerative procedures at implant placement compared to natural healing. A recent retrospective study included 418 extraction sites (171 without ARP and 247 with ARP) also showed ARP may reduce the need for bone augmentation procedure and improve the feasibility of implant placement [16]. But most of these studies merely used dental records and imaging data to evaluate the efficacy.

According to Avila-Ortiz et al. [34], peri-implant phenotype is crucial to peri-implant health, function, and esthetics. Soft tissues take over an important position in the components of peri-implant phenotype. The use of intraoral scan allows to record the profile of soft tissues without any pressure. It can be used immediately after surgical procedures without disrupting the wound. Digital scanned data make it possible to measure the dimensional changes at different time points in a long-term follow-up period [26]. Up to now, only a few studies reported the three-dimensional profilometric changes on the soft tissue level based on intraoral scanned data following tooth extraction and ARP [24–26]. In the esthetic zone of single extraction sites, Chappuis et al. [24] investigated the interplay between the soft tissue morphology and the underlying bone anatomy during an 8-week healing period by sequential digitized impressions and CBCT images. Wongpairojpanich et al. [26] used intraoral scanner for monitoring the wound closure and surface dimensional changes of alveolar ridge at different time points (1, 3, 7, 14, and 28 days and 4 months after ARP). Song et al. [25] explored the changes of soft tissue dimensions following alveolar ridge preservation and spontaneous healing in posterior maxilla using intraoral scanned data and CBCT data in a 6-month healing period. To summarize, the follow-up time for current studies was relatively short. And to the best of our knowledge, there has not been specific evidence available to identify the soft tissue contour changes of ridge preservation on molar extraction sockets with periodontal pathosis before tooth extraction.

DBBM is a type of bone substitute material widely used in dental practice [35]. A systematic review included five studies showed that ridge preservation using DBBM had a lower amount of new bone formation in comparison with natural healing, but due to the lack of randomized controlled trials, limited sample size and high heterogeneity, further experiments are needed to verify this conclusion [28]. Koo et al. [36] carried out a histological analysis at 4-month after ARP in damaged extraction sites, showing a low mean proportion of regenerated bone and a large variation from 0 to 43%. Further studies with longer healing time in periodontally compromised sockets are needed for evaluating the histological changes of ridge preservation.

This study will produce data on hard and soft tissue dimension changes at different time points and provide histomorphology evaluation following ARP and natural healing in periodontally compromised molar extraction sockets. The follow-up period of this trial will last till at least 1 year after implant loading. These results may contribute to complement the missing information for ARP in molar sites with advanced periodontitis. Results of the study will be published in peer-reviewed journals.

### Trial registration

The trial has been registered at Chinese Clinical Trial Register (ChiCTR) with the identifier number ChiCTR2200056335 on February 4, 2022. The recruitment began in March 1,2022 and will be completed in March 1,2023.

## Data Availability

Data from this research will be registered with the Chinese Clinical Trial Registry Platform. Results will be published through official peer-reviewed journals.

## Abbreviations

ARP: Alveolar ridge preservation
BI: Bleeding index
CBCT: Cone beam-computed tomography
DBBM: Deproteinized bovine bone mineral
DICOM: Digital Imaging and Communications in Medicine
GR: Gingival recession
MBL: Marginal bone loss
OHI: Oral hygiene instruction
PD: Probing depth
RCT: Randomised controlled trial
ROIs: Representative regions of interest
SRP: Supra-/sub-gingival scaling, root planning
STL: Stereolithographic
WKT: Width of keratinized tissue
